# The gut microbiota-aromatic amino acid axis in cardiovascular disease: pathophysiological roles, translational biomarkers, and therapeutic targeting

**DOI:** 10.3389/fcvm.2026.1846519

**Published:** 2026-07-29

**Authors:** Yurong Wu, Rui Xu

**Affiliations:** 1 School of Clinical Medicine, Shandong Second Medical University, Weifang, China; 2 Department of Cardiology, Jinan Central Hospital Affiliated to Shandong First Medical University, Jinan, China

**Keywords:** aromatic amino acids, cardiovascular disease, gut microbiota, phenylacetylglutamine, tryptophan metabolism, tyrosine nitration

## Abstract

**Background:**

Cardiovascular disease (CVD) remains a leading global health burden, with conventional risk factors lacking sufficient predictive power. Gut microbiota-derived aromatic amino acid (AAA) metabolites and oxidative tyrosine post-translational modifications (PTMs) have emerged as novel pathophysiological regulators of CVD, but their integrated mechanistic roles, clinical biomarker value, and therapeutic potential remain to be systematically elucidated.

**Methods:**

A review on gut microbiota-AAA axis in CVD was conducted, synthesizing mechanistic, clinical, and translational evidence of AAA metabolites (phenylalanine, tryptophan, tyrosine derivatives) and tyrosine PTMs in the pathogenesis of CVD. We also analyzed the diagnostic potential of multi-omics identified combinatorial biomarkers and the preclinical/clinical evidence for targeted therapeutic strategies.

**Results:**

Phenylacetylglutamine (PAGln) activates *α*_2_A/*α*_2_B/*β*_2_-adrenergic receptors, induces platelet hyperreactivity and myocardial injury, and is associated with increased major adverse cardiovascular events (MACE) risk. Tryptophan metabolism's pro-atherogenic kynurenine axis and gut-derived indoxyl sulfate (IS) promote endothelial dysfunction, while indole-3-propionate (IPA) exerts vasculoprotective effects. Tyrosine PTMs (sulfotyrosine, 3-nitrotyrosine) regulate leukocyte recruitment and impair endothelial enzymes, with elevated 3-nitrotyrosine predicting adverse cardiac events. The integration of combinatorial biomarkers, including PAGln, IS, Kyn/Trp ratio, 3-nitrotyrosine, and sulfotyrosine, may enhance the predictive capacity of conventional risk models, though their clinical utility requires rigorous validation across diverse populations. Gut microbiota modulation, enzymatic inhibition, and receptor antagonism show preclinical/early clinical potential for CVD intervention.

**Conclusions:**

The gut microbiota-AAA axis integrates dysbiosis, inflammation, and oxidative stress to drive CVD pathogenesis, with its metabolites and PTMs providing complementary diagnostic value. If validated, interventions targeting this axis may improve CVD risk assessment and therapeutic development.

## 1 Introduction

### 1.1 The global burden of cardiovascular disease and the traditional risk factors

Cardiovascular disease (CVD)—encompassing coronary artery, cerebrovascular, and rheumatic heart diseases—remains a leading cause of global morbidity and mortality (1, 2). While established risk factors, including dyslipidemia, hypertension, tobacco use, hyperglycemia, and pro-inflammatory mediators (3–6) contribute to progression, their predictive power is limited (3, 4). This evidence gap motivates the exploration of novel determinants, such as gut microbiota-derived metabolites (7).

### 1.2 Metabolomic perspectives on emerging drivers of cardiovascular disease pathogenesis

Contemporary progress in metabolic phenotyping continues to elucidate complex pathophysiological mechanisms and facilitate biomarker identification. Reflecting on foundational work begun over six decades ago, the seminal study by Moore's group introduced ion-exchange chromatography for amino acid quantification in 1958 (8)—a development that catalyzed seven decades of research into CVD pathways linked to essential amino acid flux. Of particular note, microbial derivatives originating from dietary aromatic amino acids (AAAs) demonstrate strengthening epidemiological correlations with cardiovascular pathology (7, 9). Modern high-resolution metabolomic platforms—especially untargeted LC-MS and NMR workflows—now prove invaluable for deconvoluting formerly unrecognized mediators in CVD pathogenesis, particularly low-molecular-weight species (below 500 Da) routinely undetected by conventional assays (10).

### 1.3 AAA metabolism: expanding from protein synthesis to bioactive mediators

AAAs—specifically phenylalanine (Phe), tyrosine (Tyr), and tryptophan (Trp)—extend beyond canonical protein-building functions to serve as biosynthetic precursors for numerous physiologically active compounds (11–13). Clinically, select AAA-derived metabolites now function as potential diagnostic indicators across multiple disease contexts (14), ranging from nutritional tyrosine's modulation of neural connectivity (15) to kynurenine pathway dynamics in inflammation and mental health (16). Accordingly, thorough characterization of AAA metabolic flux patterns and their bioactive derivatives constitutes a fundamental gateway toward next-generation biomarker identification and innovative therapeutic discovery. Recent investigations exemplified by Nemet et al.'s 2023 methodology (7) have employed stable isotope dilution mass spectrometry to simultaneously quantify over 15 AAA metabolites, including microbially modified species such as indole-3-propionic acid and p-Cresyl sulfate (PCS). These measurements revealed significant associations of specific metabolite clusters with incident major adverse cardiovascular events (MACE) and all-cause mortality—relationships that remained statistically significant after adjusting for Framingham risk scores, thereby suggesting a potential role for gut microbiome-derived AAA transformation products in cardiovascular pathogenesis.

### 1.4 Scope and novelty: integrating gut microbiota-derived metabolites with host modifications

This review summarizes recent advances in understanding the roles of AAA metabolites in CVD. It integrates insights into gut microbiota-derived AAA metabolites, such as phenylacetylglutamine (PAGln) from phenylalanine, which interacts with adrenergic receptors to modulate platelet activation, and microbial indole derivatives from tryptophan, which influence vascular inflammation. We also consider host-mediated post-translational modifications (PTMs), notably tyrosine sulfation and nitration, which alter protein function and signaling pathways relevant to vascular integrity and inflammation, elucidating their distinct and convergent mechanistic roles within the cardiovascular system. By synthesizing evidence linking these metabolites and PTMs to specific CVD pathophysiologies-including heart failure, endothelial dysfunction, atherosclerosis, and hypertension-this review evaluates available evidence on the translational potential of these metabolites and PTMs as candidate diagnostic biomarkers and therapeutic targets.

Consistent with the narrative review format, our literature synthesis was guided by a concept-adaptive search framework across PubMed and Web of Science (covering 2015–2025). Rather than applying static screening filters, we utilized widely recognized milestone studies (e.g., cell- and receptor-specific profiling studies) as foundational anchors to map cross-disciplinary knowledge networks through forward and backward citation web tracing. This approach affords perspectives for the prevention and treatment of CVD. It also highlights translational clinical implications.

### 1.5 Literature search strategy

As a narrative review synthesizing a rapidly evolving and interdisciplinary field, this article does not follow a pre-registered systematic review protocol. Nevertheless, to ensure transparency, the literature identification process is described in detail below.

The literature was identified through a multi-pronged strategy. First, a core set of landmark studies was selected based on the authors' prior expertise and continuous monitoring of the field (e.g., Nemet et al. 2020 Cell (22); Nemet et al. 2023 European Heart Journal (7); Zhu et al. 2023 Cell Host & Microbe (25). Second, forward and backward citation tracking of these anchor papers was performed using the citation tracking functions of the accessed databases to capture both foundational mechanistic studies and subsequent clinical extension research. Third, targeted searches in PubMed and Web of Science were conducted periodically between 2015 and 2025. Boolean search strings combining terms for three conceptual domains—gut microbiota (e.g., “gut microbiota,” “gut microbiome,” “dysbiosis,” “microbial metabolite”), aromatic amino acid metabolites (e.g., “phenylacetylglutamine,” “PAGln,” “indoxyl sulfate,” “kynurenine,” “3-nitrotyrosine,” “sulfotyrosine,” “tryptophan metabolism,” “phenylalanine”), and cardiovascular disease (e.g., “cardiovascular disease,” “heart failure,” “coronary artery disease,” “atherosclerosis,” “hypertension,” “MACE”)—were employed and iteratively refined throughout the review period to capture emerging concepts and newly identified metabolites. Because these strings were continuously updated rather than locked at a single time point, a single fixed Boolean query does not accurately reflect the dynamic search process. An illustrative search structure representing the three core domains is provided in Supplementary Table S1. Fourth, during the peer-review process, additional manuscripts suggested by reviewers and editors were evaluated and included where relevant. Duplicate records across different search strategies were identified and removed using EndNote. The final inclusion of 134 articles was based on relevance to the gut–AAA–CVD axis, study quality (preference was given to large prospective cohorts, well-controlled mechanistic studies, and peer-reviewed original research), and contribution to the conceptual framework of the review.

Because this is a narrative review, a formal risk-of-bias assessment was not performed. However, the level of evidence (e.g., large prospective cohort vs. animal model vs. *in vitro* study) is considered throughout the discussion, and studies with conflicting findings are noted where appropriate. The inherent limitations of this flexible, concept-adaptive approach, including potential selection bias, are acknowledged in Section 7 (Limitation).

## 2 AAA metabolic networks with cardiovascular relevance

### 2.1 Phenylalanine: host-microbial interactions in PAGln generation

Phenylalanine is an essential amino acid. Large-scale prospective population studies and metabolomic profiling have firmly established circulating phenylalanine and dietary amino acid flux as independent predictors of metabolic risk, general cardiovascular events, and incident heart failure (17–21). Phe serves as a precursor to the gut microbial metabolite PAGln, which is linked to CVD pathogenesis (22). Following dietary protein intake, phenylalanine is metabolized by gut microbiota, such as Ruminococcaceae, Lachnospiraceae, and Christensenellaceae, to produce phenylpyruvic acid (PPY) (23, 24). PPY is converted to phenylacetic acid (PAA) via two distinct thiamine pyrophosphate-dependent microbial pathways: one involving oxidative decarboxylation catalyzed by phenylpyruvate ferredoxin oxidoreductase (PPFOR), and the other involving non-oxidative decarboxylation catalyzed by phenylpyruvate decarboxylase (PPDC) (25). Subsequently, absorbed PAA is conjugated with glutamine (Gln) in the liver or kidneys through enzymatic action to produce PAGln, characterized as a metabolite participating in adrenergic signaling (22, 25).

### 2.2 Tryptophan: distinct metabolic pathways in immune and vascular interactions

Tryptophan is an essential amino acid obtained from the diet, as the body cannot produce it (26). In humans, most tryptophan is metabolized through two main pathways: the serotonin pathway and the kynurenine pathway (27). Approximately 5% of tryptophan enters the serotonin pathway, while 95% is metabolized via the kynurenine pathway (KP) (28). In the KP, enzymes called indoleamine 2,3-dioxygenase (IDO) or tryptophan 2,3-dioxygenase (TDO) convert tryptophan to N-formylkynurenine. This step is the rate-limiting step of the pathway (29–32). Next, N-formylkynurenine is enzymatically converted to kynurenine (Kyn), which leads to metabolites such as kynurenic acid, 3-hydroxykynurenine, 3-hydroxyanthranilic acid (3-HAA), and quinolinic acid (33, 34). Gut microbiota can also transform tryptophan into many indole derivatives, including indole, tryptamine, indole ethanol (IE), indole propionic acid (IPA), indole lactic acid (ILA), indole acetic acid (IAA), indole aldehyde (IAld), and indole acrylic acid (IA) (34–37). These metabolites modulate inflammation, metabolism, immune response, and neurological function by acting on receptors such as the aryl hydrocarbon receptor (AhR) and pregnane X receptor (PXR) (38).

### 2.3 Tyrosine: precursor to catecholamines and a site of pathogenic post-translational modifications

Tyrosine is a non-essential amino acid converted from phenylalanine within hepatic and renal tissues (39–42). It is the initial substrate for the synthesis of thyroid hormones, such as thyroxine (T4) and triiodothyronine (T3), which serve as pivotal hormonal modulators of cardiac contractility and vascular tone (43). Tyrosine is also a precursor for the catecholamine neurotransmitters dopamine, norepinephrine, and epinephrine (44, 45). These catecholamines modulate nervous system activity, stress adaptation, and cardiovascular homeostasis, including blood pressure and cardiac function. Besides its biosynthetic roles, tyrosine undergoes post-translational modifications (PTMs) that are significant in cardiovascular pathology. Specifically, it is subject to sulfation and nitration. Tyrosine sulfation occurs in the trans-Golgi network, where a sulfate group from 3′-phosphoadenosine-5′-phosphosulfate (PAPS) transfers to a tyrosine residue by tyrosylprotein sulfotransferases (TPSTs) 1 and 2 (46, 47). This modification regulates various biological processes, such as chemokine receptor recognition and inflammatory responses (47). Tyrosine nitration, primarily mediated by peroxynitrite and related species, produces 3-nitrotyrosine (3-NT). This can alter protein structure and function and is linked to endothelial dysfunction (48–51) (Table 1).

**Table 1:** Cardiovascular-active aromatic amino acid (AAA) metabolites and their pathophysiological roles.

Metabolite class	Representative molecules	Biosynthetic pathway	Primary molecular targets	Major pathophysiological effects	Key clinical associations
Phenylalanine derivatives	Phenylacetylglutamine (PAGln)	Gut microbial decarboxylation → hepatic hGAT conjugation	*α*_2_A/α_2_B/*β*_2_-adrenergic receptors	↑ Platelet activation, myocardial fibrosis, Ca²⁺ dysregulation	Candidate MACE risk, HF progression
Tryptophan-inflammatory	Kynurenine (Kyn)	Host IDO/TDO conversion	Aryl hydrocarbon receptor (AhR)/ROS pathways	Endothelial senescence, monocyte recruitment, plaque vulnerability	Kyn/Trp ratio: proposed CAD predictor
Microbial tryptophan derivatives	Indoxyl sulfate (IS)	Gut bacterial tryptophanase → hepatic sulfation	AhR	Endothelial-mesenchymal transition, ↓ NO bioavailability, ↑ AngII sensitivity	Context-dependent CKD hypertension biomarker
Tyrosine PTMs	3-Nitrotyrosine (3-NT)	Peroxynitrite (ONOO^−^)-mediated nitration	Prostacyclin synthase/MnSOD	Vasoconstriction, oxidative stress amplification,	Investigated ACS predictor
Tyrosine PTMs	Sulfotyrosine	TPST-mediated sulfation	PSGL-1/CCR chemokine receptors	Leukocyte adhesion ↑, thrombus formation	Associated with severe coronary stenosis

## 3 AAA metabolites in cardiovascular disease pathogenesis

### 3.1 Phenylacetylglutamine as a gut microbial mediator of heart failure through adrenergic signaling

#### 3.1.1 Clinical evidence: dose-dependent association between PAGln levels and heart failure risk and severity

As detailed in Section 2.1, PAGln biosynthesis involves gut microbial transformation of phenylalanine to phenylacetate (PAA) followed by host conjugation with glutamine (25). Clinical studies have identified a significant dose-dependent association between elevated PAGln levels and adverse cardiovascular outcomes (52). Specifically, PAGln concentrations are linked to an increased risk of major adverse cardiovascular events (MACE) and all-cause mortality in large prospective cohorts, independent of established risk factors (22). Moreover, elevated circulating PAGln levels are correlated with the presence and severity of coronary artery disease (CAD), as determined by coronary angiography. These elevated levels are also associated with the severity of heart failure (HF) and increased mortality risk in diverse patient populations, including those with chronic kidney disease (CKD), acute coronary syndrome (ACS), and chronic HF (7, 22, 23, 25, 52). These findings suggest PAGln as a potentially informative metabolite, though its clinical utility as a biomarker requires validation across diverse populations and comorbidities, particularly given that systemic aromatic and branched-chain amino acid fluxes are tightly intertwined with upstream dietary protein patterns and overlapping cardiometabolic traits, including hypertriglyceridemia and insulin resistance (53–55).

#### 3.1.2 Molecular mechanisms: modulation of adrenergic receptors and cardiodepression

PAGln significantly influences cardiovascular function by modulating adrenergic signaling pathways. High levels of PAGln contribute to MACE by activating *α*_2_A, *α*_2_B, and *β*_2_-adrenergic receptors (AdrRs) (22, 25). Studies show that PAGln activates specific G-protein-coupled receptors—*α*_2_A-, *α*_2_B-, and *β*_2_-adrenergic receptors (AdrRs) (22). This activation causes several harmful effects on the heart and blood vessels. First, it raises platelet activity and increases the risk of arterial blood clots by allowing more calcium into cells and causing granule release (22). Second, when *β*_2_-AdrRs are activated, they disrupt calcium homeostasis in heart muscle cells and promote fibrosis, increasing the likelihood of arrhythmias (56). Third, stimulation of *α*_2_A-AdrRs causes blood vessels to constrict, raising the pressure the heart must work against and increasing stress on the heart (57). This activation causes abnormalities in cardiomyocyte calcium handling, increases platelet activation and aggregation, worsens cardiac fibrosis, and triggers cardiac dysfunction in pre-clinical models (22). PAGln also increases cardiac hypercontractility *in vitro*, which may lead to arrhythmias and long-term cardiotoxicity (58, 59). Overall, these findings identify PAGln as a central link between gut microbial metabolism, increased adrenergic activity, and adverse cardiac remodeling in the development of heart failure.

#### 3.1.3 Outstanding questions: beneficial and detrimental effects, receptor subtype specificity

PAGln has been associated with adverse cardiovascular outcomes, but substantial knowledge gaps remain. It's unclear what it does under normal conditions, and any potential benefits are incompletely characterized. The molecular mechanisms underlying PAGln's interactions with specific adrenergic receptor subtypes also require clarification, as PAGln differentially affects *α*2A- and *β*2-AR signaling kinetics (22, 60). The regulation of microbial PAA production pathways and hGAT activity in host tissues is still unclear. The therapeutic value of targeting PAGln formation or signaling in HF also needs validation in clinical trials (61).

### 3.2 Tryptophan metabolic dysregulation: implications for inflammation, immunity, and vascular dysfunction

#### 3.2.1 Kynurenine pathway: IDO and TDO as inflammatory regulators in atherosclerosis

In humans, tryptophan catabolism branches into distinct physiological vectors; while a highly regulated fraction is converted into serotonin to modulate neuroendocrine and platelet functions (62), the vast majority enters the kynurenine pathway (KP) under inflammatory stress. Activation of the kynurenine pathway is mediated by indoleamine 2,3-dioxygenase (IDO) and tryptophan 2,3-dioxygenase (TDO). This activation is a contributing factor in atherosclerosis and vascular inflammation. Pro-inflammatory cytokines such as IFN-*γ* and TNF-α potently induce IDO expression (29–32). Increased kynurenine (Kyn) levels and high Kyn/Trp ratios have been associated with coronary artery disease (CAD) as candidate biomarkers (34), peripheral artery disease (PAD) (63), pulmonary hypertension (64), and HF (65), as well as non-cardiovascular pathologies including bone remodeling disorders where the pathway is intrinsically activated during osteoblastogenesis (66). Metabolites produced by IDO-mediated KP activity regulate immune responses and vascular function. Kynurenine enhances the expression of adhesion molecules VCAM-1 and ICAM-1 (67). It also facilitates monocyte recruitment, induces T-lymphocyte apoptosis, and suppresses T-regulatory cell differentiation (68). These actions promote vascular inflammation and plaque progression *in vivo*. Quinolinic acid contributes to endothelial cell senescence and dysfunction. The downstream metabolite 3-hydroxyanthranilic acid (3-HAA) exerts pro-oxidant and pro-inflammatory effects on endothelial cells (69, 70). Targeting the IDO-Kyn pathway may offer a promising therapeutic approach to attenuating vascular inflammation in atherosclerosis (71).

#### 3.2.2 Gut-derived indoles: aryl hydrocarbon receptor and pregnane X receptor signaling in vascular homeostasis and hypertension

The gut microbiota metabolizes tryptophan into a range of indole derivatives, including indole, indole-3-acetate (IAA), IPA, and indoxyl sulfate (IS) (35, 36). These metabolites serve as ligands for nuclear receptors, most notably the aryl hydrocarbon receptor (AhR) and pregnane X receptor (PXR) (37, 72, 73). Importantly, signaling through AhR and PXR significantly influences vascular tone and blood pressure regulation. Of note, accumulation of indoxyl sulfate is strongly associated with CKD, CKD-related hypertension, and cardiovascular disease. IS contributes to the development of MACE by activating the aryl hydrocarbon receptor (AhR), setting off harmful processes throughout the vascular system. Activation of AhR disrupts endothelial nitric oxide synthase (eNOS) function, reducing nitric oxide (NO) availability and leading to increased blood pressure (74). This is followed by NADPH oxidase activity, which lowers tetrahydrobiopterin (BH4) levels (75) and generates superoxide, causing oxidative damage to blood vessels. As a result, inflammatory markers such as VCAM-1 and ICAM-1 are overexpressed, promoting monocyte adhesion and accelerating atherosclerosis (76). Meanwhile, IS also triggers endothelial-to-mesenchymal transition (EndMT), leading to vascular fibrosis and making plaques more prone to rupture (38). Together, these AhR-driven changes—endothelial dysfunction, oxidative stress, inflammation, and structural remodeling—create a setting that increases the risk of thrombosis, acute coronary syndromes, and heart failure (38, 77). Moreover, activation of AhR by compounds such as IS and IAA contributes to endothelial dysfunction by reducing nitric oxide (NO) bioavailability, increasing oxidative stress, promoting vascular inflammation, facilitating endothelial-to-mesenchymal transition (EndMT), increasing arterial stiffness, and enhancing angiotensin II-induced hypertension (38, 76–78). In contrast, certain indoles, such as IPA, demonstrate beneficial effects, including AhR-independent antioxidant activity and protection of the endothelial barrier, underscoring the context-dependent roles of these metabolites (73, 79). Finally, dysbiosis-induced alterations in indole profiles are implicated in the pathogenesis of hypertension (76, 80).

#### 3.2.3 Biomarker potential and therapeutic targeting of rate-limiting enzymes

Altered tryptophan metabolism represents a potential target for further biomarker exploration (81), but broad clinical translation hinges on resolving two key limitations: ethnic variability and analytical standardization (82). Measurement of KP activation, such as kynurenine levels or the kynurenine/tryptophan ratio, as well as specific microbial indoles, such as IS, may yield clinically relevant data on inflammation, CVD risk stratification, and CKD progression. Therapeutic strategies targeting enzymes that regulate tryptophan flux are under active investigation. Inhibition of IDO has demonstrated efficacy in reducing inflammation associated with atherogenesis in pre-clinical models (71). Additionally, compounds that block pathways involved in the production of harmful indole derivatives, such as IS, or that enhance the production of beneficial metabolites, are being developed. Modulation of AhR and PXR signaling is also a potential approach to mitigate endothelial dysfunction and hypertension associated with gut dysbiosis (38, 76).

### 3.3 Tyrosine post-translational modifications as oxidative stress sensors and effectors in atherogenesis

#### 3.3.1 Tyrosine sulfation in leukocyte activation and the pathogenesis of atherosclerosis

Tyrosine sulfation is a prevalent post-translational modification (PTM) primarily mediated by tyrosylprotein sulfotransferases (TPST1 and TPST2) in the Golgi apparatus, where these enzymes catalyze sulfate transfer from the donor molecule 3′-phosphoadenosine-5′-phosphosulfate (PAPS) to specific tyrosine residues (46, 83). This essential modification stabilizes ligand structures and enhances their binding affinity to receptors expressed on leukocytes and endothelial cells (83, 84), thereby serving as a critical regulator of protein-protein interactions in inflammation and atherosclerosis. Notably, sulfation of key adhesion molecules, including P-selectin glycoprotein ligand-1 (PSGL-1) on leukocytes, enables essential tethering and rolling on activated endothelium, which initiates firm adhesion and transmigration into inflamed tissues and atherosclerotic plaques. Sulfated G-protein-coupled chemokine receptors on monocytes/macrophages are essential for directing monocyte recruitment in response to atheroma-produced chemokines, including MCP-1/CCL2 and RANTES/CCL5 (47, 84). Clinically, increased plasma concentrations of the sulfation product O-sulfotyrosine are strongly associated with coronary atherosclerosis severity and future myocardial infarction risk.

#### 3.3.2 Protein tyrosine nitration as a link between oxidative stress and endothelial dysfunction

Protein tyrosine nitration occurs when reactive nitrogen species, especially peroxynitrite (ONOO^−^), modify tyrosine residues, forming 3-nitrotyrosine (3-NT) during inflammation and oxidative stress (48, 49). This change is a stable marker of nitro-oxidative stress and tissue damage, and it can have serious effects on health. ONOO^−^ forms quickly when superoxide (O_2_^−^) reacts with nitric oxide (NO), causing tyrosine nitration that often changes how proteins work. These changes, which can either reduce or increase protein function, play a role in endothelial dysfunction, an important factor in the development of atherosclerosis (48, 50, 51).

Nitration affects important cardiovascular enzymes and proteins like prostacyclin synthase (PGIS), manganese superoxide dismutase (MnSOD), and tissue plasminogen activator (tPA). These changes together cause blood vessels to narrow, reduce the amount of available nitric oxide, increase oxidative stress, make it harder for the body to break down blood clots, and accelerate foam cell formation (48, 51).

High levels of nitrotyrosine in the blood are often found in people with heart and blood vessel diseases like coronary artery disease (CAD) (85), congestive heart failure (CHF) (86), heart attacks (AMI) (87), high blood pressure, high cholesterol, and diabetes (51). These higher levels are linked to more severe symptoms and can predict worse outcomes. Studies show that people with CAD have about three times more nitrotyrosine in their blood than healthy people (85). Higher levels in blood and urine are also linked to the frequency and severity of in-stent restenosis (ISR) (88).

#### 3.3.3 Oxidative stress-derived isomers (m-Tyr/o-Tyr): secondary contributors

In addition to enzymatic post-translational modifications (PTMs), tyrosine can be oxidized directly, primarily by hydroxyl radical (•OH) attack. This process forms the non-proteinogenic isomers meta-tyrosine (m-Tyr) and ortho-tyrosine (o-Tyr), which are mainly generated during periods of elevated oxidative stress (89, 90). High levels of m-Tyr and o-Tyr serve as reliable biomarkers of cumulative protein damage caused by free radicals *in vivo* and have been associated with increased atherosclerosis risk in epidemiological studies (91, 92). Furthermore, *in vitro* studies suggest that these oxidized tyrosine species may directly contribute to endothelial dysfunction and vascular injury, acting as secondary effectors in oxidative stress-induced cardiovascular pathology (89, 90). These changes are particularly noticeable in gut dysbiosis (Section 4.2), where oxidative stress caused by microbial metabolites increases nitration and sulfation.

## 4 Convergent pathophysiological mechanisms and clinical implications

### 4.1 Gut Microbiota as modulators of cardiovascular metabolites

The gut microbiota serves as a key metabolic regulator, significantly influencing cardiovascular risk by altering the metabolism of dietary amino acids (25, 35, 36, 44). Extensive evidence highlights its crucial role in producing metabolites linked to cardio-renal diseases (23, 25). Certain gut bacteria transform Phe, Tyr, and Trp into active compounds such as PAGln, IS, and PCS (25, 93). When gut microbial composition changes—a state known as dysbiosis—the balance between harmful and beneficial metabolites, like IPA, shifts (7, 38). This shift affects vulnerability to hypertension, atherosclerosis, heart failure, and cardiovascular complications related to CKD (22, 76, 78). Microbial metabolites such as PAGln and IS interact with host receptors, including G protein-coupled receptors (GPCRs) and the aryl hydrocarbon receptor (AhR), to trigger cellular responses. Together, these findings position the gut microbiome as a crucial and modifiable environmental factor in cardiovascular health (22). As discussed in Section 4.2, dysregulation also affects post-translational modifications as a result of oxidative stress propagation.

### 4.2 Gut dysbiosis as an amplifier of tyrosine-driven cardiovascular pathology through oxidative stress cascades

Gut dysbiosis increases oxidative stress in the body via three main pathways that, together, worsen harmful tyrosine modifications (94). Pro-oxidative microbial metabolites lower endothelial glutathione levels and disrupt mitochondrial electron transport, thereby increasing superoxide (O_2_•^−^) production (95). Meanwhile, NADPH oxidases (including NOX2 and NOX4) serve as critical cellular enzymatic sources primarily responsible for the regulated generation of reactive oxygen species (ROS) under diverse pathological stimuli (96). The problem worsens when gut-derived uremic toxins block Nrf2-driven antioxidant gene expression and reduce cellular ascorbate and *α*-tocopherol levels (97), weakening the body's redox defenses (98).

This oxidative state speeds up the formation of peroxynitrite (ONOO^−^) from superoxide (O_2_•^−^) and nitric oxide, which causes harmful tyrosine nitration in prostacyclin synthase (PGIS) and manganese superoxide dismutase (MnSOD). These changes permanently damage the protective and antioxidant roles of these proteins (48, 51). At the same time, the modification of chemokine receptors by tyrosylprotein sulfotransferases (TPST1/2) increases sulfotyrosine-driven leukocyte adhesion and clotting; this process actively triggers NF-*κ*B activation and fuels oxidative stress-related inflammation (99). These damaging changes persist longer when the body cannot remove nitrotyrosine-damaged proteins effectively, especially in kidney disease, which prolongs vascular injury (100).

Clinical data have reported associations between reduced Faecalibacterium prausnitzii and increased Enterobacteriaceae and markers of vascular inflammation and systemic oxidative stress in patients with CKD and CVD (101, 102). These observations are consistent with a link between gut microbiota alterations and oxidative stress–driven vascular pathology, further supporting the concept of the gut-AAA-PTM axis.

### 4.3 Post-translational modifications as amplifiers of oxidative and inflammatory injury

Protein tyrosine modifications act as key amplifiers of vascular damage. They integrate oxidative and inflammatory insults into sustained functional impairment (103). Tyrosine sulfation is catalysed by TPSTs in the Golgi apparatus (46, 83). This process regulates leukocyte trafficking and the function of adhesion molecules, such as PSGL-1 and CCRs (47, 84). As a result, it directly contributes to inflammatory cell infiltration in atherosclerosis (47, 103). At the same time, pathological tyrosine nitration produces 3-nitrotyrosine (3-NT) and is driven by peroxynitrite (ONOO^−^) during oxidative stress. This nitration impairs important vascular proteins, including prostacyclin synthase (PGIS), manganese superoxide dismutase (MnSOD), and tissue plasminogen activator (tPA) (48, 51, 104, 105). The resulting nitration cascade leads to endothelial dysfunction, vasoconstriction, impaired fibrinolysis, and foam cell formation (48, 51, 85). This complex pro-thrombotic milieu is further compounded by tryptophan-derived serotonin signaling, which has been shown to aggressively enhance platelet procoagulant properties and accelerate vascular activation cascades (106). In addition to enzymatic PTMs, oxidative stress generates non-enzymatic tyrosine isomers (m-Tyr, o-Tyr). These serve as stable indicators of cumulative free radical damage and correlate with atherosclerotic risk (85). In summary, covalent tyrosine modifications act as persistent markers of oxidative and inflammatory stress, translating initial insults into chronic vascular pathology (104, 105, 107).

### 4.4 Multi-omics strategies for biomarker discovery

Integrative multi-omics platforms, such as metabolomics (81) and proteomics (88), can identify candidate biomarkers that may become clinically actionable after robust validation (108). These biomarkers reflect gut microbial metabolic activity and oxidative stress. Targeted plasma metabolomics allows precise measurement of gut microbiota-derived uremic toxins (PAGln, IS, PCS), oxidized amino acids (m-Tyr, o-Tyr), post-translational modification biomarkers (nitrotyrosine, sulfotyrosine), and metabolites from the kynurenine pathway (22, 109–111). Integrating plasma microbial metabolite levels (e.g., PAGln, IS) with host genetic risk scores may provide complementary value in specific cohorts, though multicenter validation is needed to confirm generalizability (112). Integrating metabolomic signatures (like the Kyn/Trp ratio) with proteomic or inflammatory marker profiles may enhance stratification in CVD (65). It helps predict outcomes, including in-stent restenosis (ISR) and heart failure severity (52, 113). While multi-omics approaches represent potential tools for risk stratification, their clinical utility requires robust validation in diverse populations to address performance variability (114).

### 4.5 Therapeutic strategies: targeting enzymatic, receptor, and microbial pathways

Emerging therapeutic strategies to intercept AAA metabolite-mediated pathophysiology operate at complementary biological levels: At the biosynthetic level, inhibiting rate-limiting enzymes—such as intestinal bacterial decarboxylases/precursor transporters to suppress phenylacetic acid (PAA, precursor to PAGln) (25), indoleamine 2,3-dioxygenase (IDO) to reduce pro-atherogenic kynurenine (Kyn) production (115), or tyrosylprotein sulfotransferases (TPSTs) to modulate leukocyte recruitment (46, 47)—disrupts pathological metabolite generation. Simultaneously, at the effector level, pharmacological agents antagonize metabolites through receptor blockade/modulation, exemplified by *β*-blockers and *α*2-adrenergic receptor (*α*2-AR) antagonists that counteract PAGln-mediated effects, as well as aryl hydrocarbon receptor (AhR) antagonists that mitigate IS toxicity (22, 38, 116). Upstream microbial reprogramming leverages prebiotics, probiotics, dietary adjustments (e.g., resistant starch/fiber) that may theoretically synergize with pharmacological agents such as *β*-blockers, targeted bacteriophages, or fecal microbiota transplantation (FMT) to repress bacteria producing detrimental metabolites (e.g., PAA-, IS-, PCS-generating species) while enriching beneficial taxa, such as *Clostridium sporogenes* (producer of IPA) (25, 76, 117, 118) (Table 2). Collectively, this multi-tiered approach—encompassing enzymatic pathway suppression, receptor engagement blockade, and gut microbial ecology modulation—provides a rationale for further clinical evaluation. Although preclinical strategies, such as IDO inhibition in atherogenesis models and AhR antagonism in CKD-hypertension, show mechanistic proof of concept, clinical diagnostic applications necessitate rigorous validation of specificity and standardized biomarkers.

**Table 2:** Therapeutic strategies targeting the Gut-AAA-CVD axis.

Intervention level	Approach	Exemplary agents/tools	Molecular target	Evidence status
Microbiome modulation	Prebiotics	Resistant starch	Enrich IPA-producers	Animal validated (119)
	FMT	Healthy donor microbiota	Suppress PAA/IS-generating bacteria	Phase I clinical trial (120)
Enzymatic inhibition	IDO blockers	Epacadostat, 1-MT	Kynurenine production ↓	Atherosclerosis models (71)
	Decarboxylase inhibitors	α-Fluorophenylalanine	PAGln precursor blockade	*In vitro* confirmation (25)
Receptor antagonism	AhR inhibitors	CH-223191, StemRegenin 1	IS toxicity neutralization	CKD-hypertension models (116)
	α_2_-AR antagonists	Yohimbine	PAGln signaling blockade	Cardiomyocyte studies (22)
Integrated therapy	Diet-drug combos	High-fiber + β-blockers	Theoretical metabolic homeostasis	Hypothetical strategy (No direct evidence)

## 5 Future perspectives

### 5.1 Mechanistic gaps in cell-type-specific signaling and receptor isoform selectivity

Resolving cell- and receptor subtype-specific signaling effects of gut microbiota-derived metabolites represents a critical frontier. Current evidence suggests divergent responses to metabolites like PAGln and IS across human endothelial cells, macrophages, vascular smooth muscle cells (VSMCs), and platelets (22, 76). However, isoform-selective activation of adrenergic receptors (e.g., *α*_2_A vs. *α*_2_C-AR subtypes for PAGln), AhR/*β*-arrestin-mediated non-genomic signaling for IS, and tissue-specific G-protein-coupled receptor (GPCR)/solute carrier (SLC) engagement mechanisms remain incompletely characterized (60, 116). Elucidating these pathways is essential for predicting off-target effects and developing cell-targeted therapeutics.

### 5.2 Establishing causality through Mendelian randomization and microbial transplantation

To clearly establish whether microbial metabolites contribute to human cardiovascular disease, researchers need to use both advanced epidemiological methods and experimental studies. Bidirectional Mendelian randomization (MR) studies that use genetic variants in metabolite-associated genes—such as POTEB and ABO for PAGln and UGT1A and SLC22A for uremic toxins—provide stronger evidence for causality compared to observational studies, which only show associations between elevated PAGln, PCS, and IS and thrombotic or atherogenic phenotypes (7, 22). Additionally, microbial transplantation studies utilizing fecal microbiota transfer from human donors to gnotobiotic rodent models colonized with defined bacterial consortia are critical for isolating the effects of specific microbial metabolic pathways. This approach is particularly important for metabolites without enzymatic synthesis analogues in mice, such as PAGln (25).

### 5.3 Model translation: human-relevant systems

Developing disease models that closely mimic human biology is crucial for translating research findings. Established approaches include primary human cell co-cultures, vascular organoids, and patient-derived inducible pluripotent stem cell (iPSC) models (121). Incorporating hemodynamic shear stress and immune components can help bridge mechanistic discoveries to human physiology. Multi-omics data—including metagenomics, metabolomics, and host transcriptomics—can be integrated into gnotobiotic mouse models humanized by defined gut microbiota inoculation (25). This enables functional validation of specific bacterial species or gene clusters, such as tyrosine decarboxylases (25) and indole-producing genes (76), in metabolite-driven pathologies. These models also permit investigation of dose-dependent and metabolite-combination effects (22).

### 5.4 Clinical translation: standardization of biomarker assays and early-phase trials

Effective clinical translation depends on standardizing biomarker methodologies and implementing early-phase therapeutic trials. Harmonization of mass spectrometry protocols for quantifying microbial metabolites (such as PAGln, IS, and PCS), oxidized amino acids (m- and o-tyrosine), and post-translational modification biomarkers (nitrotyrosine and sulfotyrosine) across laboratories is essential for ensuring that these biomarkers can be reliably evaluated in clinical validation studies and, if successful, considered for diagnostic use (122, 123). Furthermore, Phase 0/I pharmacodynamic trials evaluating inhibitors that target AAA metabolic pathways, including indoleamine 2,3-dioxygenase (IDO) inhibitors, aryl hydrocarbon receptor (AhR) antagonists (such as CH-223191 and StemRegenin 1), bacterial decarboxylase blockers, and PAGln GPCR signaling antagonists, are necessary to assess target engagement, safety, and early biomarker modulation (for example, Kyn/Trp ratio and IS clearance) (124, 125).

### 5.5 Systems biology: integration of host genetics, microbial metagenomics, and metabolomics

Maximizing therapeutic opportunities requires integrated multi-scale modeling. Computational frameworks that combine host genomic risk scores, longitudinal functionally resolved metagenomic sequencing, comprehensive plasma and urinary metabolomics, and clinical outcomes data (such as MACE, ISR, and plaque vulnerability) remain underutilized (7, 126). AI-driven integrative approaches have the potential to identify high-risk patient clusters, such as specific metabotypes characterized by gut dysbiosis signatures (127), elucidate microbe–host–diet–drug interactions influencing metabolite production, and predict responses to dietary or mineralocorticoid receptor antagonist interventions for disease interception in pre-symptomatic individuals (128).

## 6 Assessment of knowledge gaps and translational barriers

Gut-derived AAA metabolites are associated with the risk of CVD, but there are practical challenges to using them in clinics. Biomarkers such as PAGln, indoxyl sulfate, and sulfotyrosine may aid in risk assessment when integrated with traditional risk factors. However, the idea that these are complementary to traditional biomarkers needs stronger evidence. One major issue is limited specificity. For example, elevated PAGln levels were observed in CKD even in the absence of CVD (129), making it difficult to draw clear conclusions. Ethnic differences also matter. Research found that the kynurenine/tryptophan (Kyn/Trp) ratio predicts outcomes better in Asian groups than in the European population groups (130), making it difficult to apply these findings to everyone. Studies show these biomarkers have only moderate effectiveness. This suggests these biomarkers should support, not replace, current risk assessment tools (131).

Differences between preclinical and clinical results also slow down the development of new treatments. For example, PAGln activates *β*_2_-adrenergic receptors in human platelets, but this effect does not occur in mice (22). In another case, indoleamine 2,3-dioxygenase (IDO) inhibitors reduce atherosclerosis in mice, but human trials, such as the Phase III epacadostat study in cancer (132), have shown little benefit. These examples show how biological differences between species can limit the extent to which findings from animal models translate to humans. Consequently, while population studies demonstrate epidemiological associations, translating these findings into clinical diagnostics necessitates standardization across ethnic groups and validation in multi-center research (7).

The implementation of microbiome-based CVD diagnostics is hindered by several practical challenges. Microbial metabolites exhibit considerable instability; for example, PAGln levels can fluctuate in response to dietary changes, complicating consistent analysis (25). In addition, metabolomic screening is expensive, so these tests are likely to be used alongside existing methods rather than replacing them (133). Because of these challenges, it makes sense to add microbiome-based tools gradually to current CVD risk assessments rather than adopt them fully right now (134).

## 7 Limitation

As a narrative review of an interdisciplinary topic, this work adopts a flexible literature selection strategy to balance comprehensiveness and conceptual coherence, which differs from the strictly pre-specified protocol of systematic reviews. This approach may introduce potential selection bias, and readers should consider this when interpreting the synthesized evidence.

## 8 Conclusion

Gut-derived AAA metabolites and oxidative PTMs emerge as critical pathophysiological hubs. These molecular signatures integrate gut dysbiosis with vascular inflammation through receptor-mediated pathways (GPCRs/AhR) and oxidative protein modifications. Integrated omics approaches identify novel biomarker candidates (PAGln, IS, etc.) that may serve as candidate or complementary tools for conventional risk assessment, only after rigorous validation of analytical standardization, population-specific performance, and cost-effectiveness. Potential therapeutic strategies include microbiome modulation (prebiotics/FMT/bacteriophages), enzyme inhibition (decarboxylases/IDO/TPSTs), and receptor targeting (AhR antagonists/*α*_2_-AR blockers).

Future directions demand multipronged advances. Primarily, elucidation of cell-specific signaling—*α*_2_A- vs. *α*2C-AR distinction, AhR/*β*-arrestin pathway mapping—remains imperative. Concurrently, causal validation requires Mendelian randomization, gnotobiotic models, and human-relevant platforms (vascular organoids/microbiome-humanized mice). Ultimately, a validated understanding of the gut-AAA-PTM axis could contribute to refined CVD risk stratification in specific subpopulations, provided that findings are validated in large cohorts and their cost-effectiveness is assessed.
