# Supplementary material for: The gut microbiota-aromatic amino acid axis in cardiovascular disease: pathophysiological roles, translational biomarkers, and therapeutic targeting
Source: Front Cardiovasc Med. 2026 Jul 29;13:1846519. doi: 10.3389/fcvm.2026.1846519 (PMC13462210; doi:10.3389/fcvm.2026.1846519)
Supplement: Supplementary file 1 [file Table1.docx]

**Supplementary Table 1. Boolean Search Strategy**

| **Domain** | **Representative Boolean Query (PubMed format)** |
| --- | --- |
| Gut microbiota | (“gut microbiota”[Title/Abstract] OR “gut microbiome”[Title/Abstract] OR “dysbiosis”[Title/Abstract] OR “microbial metabolite”[Title/Abstract]) |
| Aromatic amino acid metabolites | (“phenylacetylglutamine”[Title/Abstract] OR “PAGln”[Title/Abstract] OR “indoxyl sulfate”[Title/Abstract] OR “kynurenine”[Title/Abstract] OR “3-nitrotyrosine”[Title/Abstract] OR “sulfotyrosine”[Title/Abstract] OR “tryptophan metabolism”[Title/Abstract] OR “phenylalanine”[Title/Abstract]) |
| Cardiovascular disease | (“cardiovascular disease”[Title/Abstract] OR “heart failure”[Title/Abstract] OR “coronary artery disease”[Title/Abstract] OR “atherosclerosis”[Title/Abstract] OR “hypertension”[Title/Abstract] OR “MACE”[Title/Abstract]) |

Note: The above queries were combined with AND operators and iteratively refined throughout the review period. Additional terms (e.g., “indole-3-propionate,” “p-Cresyl sulfate,” “tyrosine nitration”) were added as the field evolved and new evidence emerged. This table represents the conceptual structure rather than a single fixed query applied at any one time point.
